# Ending the TB epidemic – what can we learn from the HIV epidemic?

**DOI:** 10.5588/ijtldopen.24.0618

**Published:** 2025-02-01

**Authors:** A.D. Harries

**Affiliations:** International Union Against Tuberculosis and Lung Disease, Paris, France; London School of Hygiene & Tropical Medicine, London, UK.

**Keywords:** tuberculosis, ART, active case-finding, AIDS

## Abstract

Advocates for community-wide screening promote this approach to reduce TB incidence and end the TB epidemic. The roll-out of antiretroviral therapy (ART) for HIV-infected people is often cited as evidence that such ambitious programmes can work, even in remote, low-resource settings. It is important to remember that three landmark interventions made the HIV/AIDS response so successful. These were: simple-to-use tests made point-of-care HIV testing universally available; safe, acceptable, effective ART saved lives; and pre-exposure prophylaxis prevented transmission. A similar level of diagnostic simplicity and well-tolerated treatments need to be developed to make community-wide screening for TB affordable, scalable and effective.

We are woefully short of achieving the targets for ending the TB epidemic. According to the Sustainable Development Goals, these are a 90% reduction in the number of TB deaths and an 80% reduction in TB incidence in 2030 compared to 2015.^1^ Key to achieving these targets are early diagnosis and treatment of TB (including drug-resistant TB), systematic screening of contacts and high-risk groups, management of comorbidities (including HIV), and preventive therapy of persons at high risk. Unfortunately, progress has been slow, and there is a growing consensus that a more effective response will only be achieved through enhanced case finding. In high TB burden areas, most TB transmission occurs in the community, outside of the household of index pulmonary TB patients.^2^ To break transmission, it has been proposed that we screen entire populations for TB and treat all those testing positive, regardless of whether they have symptoms or not. As a demonstration of this approach, a cluster randomised controlled trial was conducted in Vietnam: three years of community-wide screening resulted in a 44% lower prevalence of TB infection in children in the fourth year compared to passive case detection alone.^3^ This screening intervention was highly resource-intensive, involving house-to-house surveys, sputum specimen collection from all participants (regardless of symptoms), sputum analysis by Xpert MTB/RIF, and additional chest radiography for those testing positive. Further analysis of the data showed that this approach not only reduced the prevalence of TB infection but also the incidence of TB disease.^4^ The key question is how affordable and scalable this approach might be for national TB programmes in low- to middle-income countries (LMICs) with a high TB burden. Supporters of such ambitious programmes often cite the success of the roll-out of antiretroviral therapy (ART) for people living with HIV (PLHIV).^5^ However, we need to assess whether this is a valid comparison and consider what made the response to HIV/AIDS such a success.

## Lessons from the interventions for HIV/AIDS

AIDS was declared a pandemic in 1981. In 2005, at its peak, and before ART roll-out had got traction, an estimated 4.9 million people became newly infected with HIV and 3.1 million people died of AIDS.^6^ Fast forward 18 years to a dramatic improvement – in 2023, an estimated 1.3 million people were newly infected with HIV and 630,00 people died of AIDS.^7^ Although still tragically high, the decline in morbidity and mortality due to HIV has been remarkable, and three landmark interventions have contributed to this. The first is the diagnosis of HIV infection. The initial ELISA antibody tests for HIV diagnosis, which required laboratory infrastructure and trained laboratory personnel, were replaced by inexpensive, sensitive, specific, rapid and simple-to-use test strips. Point-of-care HIV testing could thus be undertaken by non-laboratory-based personnel in peripheral health facilities and in the community, allowing decentralisation and massive scale-up of HIV diagnosis.^8^ Self-testing also emerged as a useful and supplementary way of increasing the proportion of people with known HIV status.^9^ The second vital intervention is the provision of safe, well tolerated and effective ART. Triple combination therapy was first shown to be effective in reducing morbidity and mortality in patients with advanced HIV infection in high-income countries in the mid-1990s.^10^ In the following two decades, the costs of therapy plummeted, and the WHO-recommended regimen (at that time of tenofovir-lamivudine-efavirenz) taken as a single pill once a day, was effective, safe and non-toxic. Further work on improving the efficacy and safety of ART regimens (including the incorporation of dolutegravir in the first-line treatment regimen) paved the way for WHO to recommend that ART be offered to all PLHIV regardless of clinical stage or CD4 cell count.^8^ Not only do current ART regimens provide a near-normal life expectancy for most PLHIV who adhere to treatment, but they also prevent viral transmission to uninfected sexual partners. If the HIV-RNA viral load is undetectable, transmission of HIV does not occur, leading to the important slogans ‘treatment is prevention’ and ‘undetectable equals untransmittable’.^11^ The third landmark intervention is the use of ART (usually tenofovir and emtricitabine) as pre-exposure prophylaxis (PrEP) for people who are uninfected but at risk. A large body of evidence has accumulated to show that a once-daily single PrEP pill taken before sexual exposure is 99% effective in preventing the acquisition of HIV.^12^

These health-related technologies, combined with stigma reduction strategies, mitigation of harmful social determinants and sufficient funding, enabled UNAIDS to set the 95-95-95 targets (95% of all PLHIV should be diagnosed; of these, 95% should be on ART; and of these 95% should have viral suppression). Modelling studies suggest that meeting these targets will enable the goal of ending HIV/AIDS: considered by the United Nations to be a 90% reduction in new HIV infections and AIDS-related deaths by 2030 compared with 2010.^13^ Progress towards these global targets is steady, but variable, although several high HIV-burden countries in Africa have already surpassed these targets or are close to achievement.^14^

## Tools for TB control

How do the tools currently available for TB diagnosis, treatment and prevention measure up to those used in the HIV/AIDS response?

## Tools for TB diagnosis

Laboratory-based sputum smear microscopy has been the mainstay of TB diagnosis for over 100 years. However, due to its relative insensitivity and inability to distinguish between drug-susceptible and drug-resistant organisms, this has been largely replaced by rapid molecular diagnostic tests. One of the most frequently used is the Xpert MTB/RIF assay (GeneXpert: Cepheid, Sunnyvale, CA, USA), which allows for the simultaneous detection of Mycobacterium tuberculosis and rifampicin resistance.^15^ Although much more sensitive and specific than smear microscopy, GeneXpert remains laboratory-based, and its use in LMICs is often compromised by unstable electricity, inadequate maintenance and interrupted cartridge supplies.^16^ Simple point-of-care diagnostic tests for TB are not yet available for routine use. Cepheid’s portable, battery-operated, point-of-care version (GeneXpert Omni) has been repeatedly pushed back due to technical challenges,^17^ and lipoarabinomannan (LAM) measured in urine samples by a test strip is only really suitable in routine settings for PLHIV who are severely immunosuppressed.^16^

## Treatment regimen for TB

Over 95% of patients have drug-susceptible TB and are treated with the 6-month rifampicin-isoniazid-based regimen. Although highly effective, a substantial proportion (25–60%) of patients experience adverse events, the most common being gastrointestinal and cutaneous, while some may develop drug-induced liver injury, which can be fatal.^18^ Treatment of drug-resistant TB has undergone a dramatic transformation in the past 10 years, with WHO now recommending a 6-month oral regimen of bedaquiline, pretomanid, linezolid and moxifloxacin (BPaLM).^19^ While much more efficacious and better tolerated than the previous 9-to-20-month treatment regimens, nevertheless, at least one in four patients experience important adverse events such as QTcF prolongation, peripheral neuropathy or myelosuppression.^20^

## Preventive TB treatment

A variety of regimens are now available to prevent TB in those at risk of contracting the disease, including 6–9 months daily isoniazid, 3-months daily rifampicin-isoniazid, 3-months weekly rifapentine-isoniazid and 6-months daily levofloxacin, the latter for contacts of MDR/RR-TB.^21^ Since the 2018 United Nations High-Level Meeting (UNHLM) on TB, preventive therapy for those most at-risk of TB (PLHIV and household contacts of index pulmonary TB patients) has begun to be scaled up, although uptake against global targets is still poor mainly due to clinic visit costs, reluctance of asymptomatic contacts to consult medical officers and fears of drug-related side effects.

## CONCLUSION

The simple diagnostic tools and safe, well-tolerated treatments developed for HIV/AIDS are not yet available for TB control. There are two important steps we need to take before we can realise the dream of community-wide screening as a routine means of ending the TB epidemic. First, a lot more work is needed to simplify TB diagnosis and make TB treatments less toxic. This requires much better commitment and financial support from donors than is currently the case.^22^ For example, innovative COVID-19 point-of-care diagnostic tests that might be suitable for TB diagnosis need to be fast-tracked, and (if they work) quickly deployed in the field.^23^ Better tolerated anti-TB drugs also need to be developed. In community-wide screening, and similar to what is found in national TB prevalence studies, about one half of people with bacteriologically confirmed TB may be asymptomatic,^3,24^ The willingness of such patients to initiate and complete several months of treatment is highly dependent on non-toxic medication. The second necessary step is the need for further community-wide research, an example being the active case finding and prevention studies in Kiribati to eliminate TB and leprosy,^25^ to confirm the findings in Vietnam^3^ and strengthen the evidence base.

The clock is ticking and there is no time to be lost!
